# GDNet-EEG: An attention-aware deep neural network based on group depth-wise convolution for SSVEP stimulation frequency recognition

**DOI:** 10.3389/fnins.2023.1160040

**Published:** 2023-04-13

**Authors:** Zhijiang Wan, Wangxinjun Cheng, Manyu Li, Renping Zhu, Wenfeng Duan

**Affiliations:** ^1^The First Affiliated Hospital of Nanchang University, Nanchang University, Nanchang, Jiangxi, China; ^2^School of Information Engineering, Nanchang University, Nanchang, Jiangxi, China; ^3^Industrial Institute of Artificial Intelligence, Nanchang University, Nanchang, Jiangxi, China; ^4^Queen Mary College of Nanchang University, Nanchang University, Nanchang, Jiangxi, China; ^5^School of Information Management, Wuhan University, Wuhan, China

**Keywords:** group depth-wise convolution, EEG attention, SSVEPs, stimulation frequency recognition, EEG signal

## Abstract

**Background:**

Steady state visually evoked potentials (SSVEPs) based early glaucoma diagnosis requires effective data processing (e.g., deep learning) to provide accurate stimulation frequency recognition. Thus, we propose a group depth-wise convolutional neural network (GDNet-EEG), a novel electroencephalography (EEG)-oriented deep learning model tailored to learn regional characteristics and network characteristics of EEG-based brain activity to perform SSVEPs-based stimulation frequency recognition.

**Method:**

Group depth-wise convolution is proposed to extract temporal and spectral features from the EEG signal of each brain region and represent regional characteristics as diverse as possible. Furthermore, EEG attention consisting of EEG channel-wise attention and specialized network-wise attention is designed to identify essential brain regions and form significant feature maps as specialized brain functional networks. Two publicly SSVEPs datasets (large-scale benchmark and BETA dataset) and their combined dataset are utilized to validate the classification performance of our model.

**Results:**

Based on the input sample with a signal length of 1 s, the GDNet-EEG model achieves the average classification accuracies of 84.11, 85.93, and 93.35% on the benchmark, BETA, and combination datasets, respectively. Compared with the average classification accuracies achieved by comparison baselines, the average classification accuracies of the GDNet-EEG trained on a combination dataset increased from 1.96 to 18.2%.

**Conclusion:**

Our approach can be potentially suitable for providing accurate SSVEP stimulation frequency recognition and being used in early glaucoma diagnosis.

## 1. Introduction

Glaucoma is one of the leading causes of blindness in the world. The damage to visual function caused by glaucoma is irreversible, and it can be difficult for the patients to realize this disease until their vision is damaged. According to the World Health Organization (WHO), the number of people living with glaucoma worldwide reached 76 million in 2020 and will rise to 95.4 million by 2030 (Guedes, 2021). China is one of the countries with the largest number of glaucoma patients. In 2020, the number of glaucoma patients in China reached 21 million, of which 5.67 million were blind (Soh et al., 2021). Glaucoma is generally not preventable, but most patients can maintain adequate vision in later life if detected early and appropriately treated. Therefore, early detection and diagnosis are significant for glaucoma blindness prevention. Traditional methods for assessing functional loss in glaucoma always adopt standard automated perimetry (SAP), which requires considerable subjective response from patients. The subjective assessment is limited by large test-retest variability, and may result in late diagnosis or delayed detection of progressive degeneration of retinal ganglion cells (RGCs).

Steady-state visual evoked potentials (SSVEPs) are typically recorded by electroencephalography (EEG) and reliably applied to brain-computer interface systems (BCIs). When exposed to a fixed frequency of visual stimuli, the brain’s visual cortex produces a continuous frequency-dependent response (Nuzzi et al., 2018). This response known as SSVEPs can be used to assess functional abnormalities in visual pathways (Geethalakshmi et al., 2022). For glaucoma patients, due to the loss of peripheral vision, some constant frequency of repeated stimuli can no longer be received, so the corresponding stimulation frequency cannot be detected from the EEG brain signal (Lin et al., 2015; Chen et al., 2021, 2022a,b). Therefore, SSVEP can be considered as an objective assessment of visual field damage caused by glaucoma. For example, Lin et al., 2015 hypothesized that a brain region corresponding to a visual field deficit would be less perceivable and thereby would result in weaker SSVEP amplitude. Their study demonstrated that the SSVEP dynamics in terms of amplitude is capable of serving as objective biomarkers to assess visual field loss in glaucoma. Medeiros et al., 2016 produced nGoggle, a portable brain-based device, to assess the visual function deficits in glaucoma. Moreover, Nakanishi et al., 2017 investigated the ability of nGoggle equipment to discriminate glaucomatous from healthy subjects in a clinic-based setting. The aforementioned studies demonstrate the feasibility of using SSVEP signal to provide objective assessment of visual field damage.

The SSVEPs-based early detection for glaucoma requires effective analysis methods for recognizing stimulation frequencies. Traditional analysis methods for SSVEP signal can be mainly divided into two categories: spatial-spectral-temporal (SST) based method (Mora-Cortes et al., 2018; Salelkar and Ray, 2020; Zhang et al., 2020) and canonical correlation analysis (CCA) based method (Liu Q. et al., 2020; Cherloo et al., 2022; Ma et al., 2022). The former tries to extract SST features from the EEG signal and use them to execute classification tasks. Based on statistical analysis, the latter attempts to identify and measure the associations between the SSVEP signal and reference signal (e.g., sinusoidal signal). For example, Chen et al. (2015) construct the filter bank CCA (FBCCA) which decompose SSVEPs into multiple sub-band components under multiple pre-processing filters, then fuse the classifications from all sub-band. Although both achieve satisfactory results in SSVEPs-based applications, they require manually predefined algorithms based on expert knowledge to extract handcrafted features. This procedure is not flexible and may limit the usage of the method in SSVEPs-based applications. In recent years, convolution neural network (CNN) based deep learning (DL) methods have been widely used in processing SSVEPs-based frequency recognition tasks and achieved excellent performance (Khok et al., 2020). Combined with existing methods (e.g., SST analysis, CCA), CNN models use multiple layers to progressively extract higher-level features from model input and perform automatic feature extraction. Many advanced CNN-based technologies have been proposed in the recent years. For example, Li et al., 2022 proposed DSCNN, a dilated shuff CNN model for actualizing EEG-based SSVEP signal classification. Yao et al., 2022 constructed FB-EEGNet by fusing features of multiple neural networks for SSVEP target detection. To achieve reasonable model architecture with superior model performance, many studies designed the deep learning models specifically suited to the domain of EEG-based SSVEP signal classification. For example, Waytowich et al., 2018 proposed a Compact-CNN for classifying asynchronous SSVEPs. The Compact-CNN’s architecture is similar to EEGNet (Lawhern et al., 2018), which performs two convolutional steps (temporal convolution and depth-wise convolution) sequentially to learn frequency and frequency-specific spatial filters, respectively. Guney et al., 2021 designed a novel deep neural network (DNN) to process the multi-channel SSVEP with convolutions across sub-bands of harmonics, channels, and time and classify them at a fully connected layer. Li et al., 2020 implemented a CNN-based non-linear model, i.e., convolutional correlation analysis (Conv-CA), which first uses CNNs at the top of a self-defined correlation layer. Further, it utilizes the correlation layer to calculate the correlation coefficients between EEG and reference signals.

Previous studies of CNN-based SSVEP stimulation frequency recognition (Waytowich et al., 2018; Li et al., 2020; Guney et al., 2021) have usually adopted one-dimensional (1D) temporal convolution to mimic a bandpass frequency filter for filtering the signal of each EEG channel, followed by depth-wise spatial convolutions to combine the channels to obtain a better frequency pattern. Because the same 1D convolutional filter filters the data of each EEG channel, different rows in the same feature map contain the same EEG frequency components. The following depth-wise spatial convolution is used to learn spatial filters for each temporal filter, enabling the efficient extraction of frequency-specific spatial filters. However, the brain signal generated from different regions presents different harmonics in the same period (Atasoy et al., 2016; Retter et al., 2021), the frequency-specific spatial characteristics might be insufficient to reflect the diversity of brain signals in different brain regions. In addition, regional neural complexity and network functional connectivity may relate to the brain’s information processing (McDonough and Nashiro, 2014). The regional neural complexity reflects the richness or diversity of brain signals in different brain regions, the more complex the regional neural activity, the higher functional connectivity this region has with other brain regions. Thus, it is reasonable to believe that diverse frequency combinations across different EEG channels may play an essential role in EEG-based brain activity classification. To simulate the regional characteristics of the EEG signal and reflect the diversity, we are interested in creating the different rows in the single feature map containing different frequency components. This motivates us to use different convolutional filters to process the EEG signal of different EEG channels.

Our brain is a coherent information processing system integrated by distributed and specialized networks (Ferraro et al., 2018). The current theory of brain functional networks suggests that the integration of specialized networks in the brain is facilitated by a set of essential nodes (Shine et al., 2016; Ferraro et al., 2018). The theory highlighted the significance of specialized networks and the relation between different specialized networks in evaluating brain function. Instead of using the connectivity of all brain regions, the connectivity features of partial brain regions might be more effective in representing different brain activities accurately. However, most existing combination studies of the DL and brain functional connectivity (BFC) focus on automatically learning the global connectivity feature of all brain regions (Babaeeghazvini et al., 2021; Avberšek and Repovš, 2022; Lin et al., 2022). Few concentrate on automatically learning the local connectivity features of specialized networks and the relations between different specialized networks. Considering different brain states involve different functional connectivity networks, we have reasons to believe the EEG characteristics over the local BFC network may contain useful classification information for discriminating different brain activities. The critical step of learning specialized network characteristics by the CNN model is identifying essential nodes. The attention mechanism (Vaswani et al., 2017; Lv et al., 2021) provides an automatic solution to identify essential nodes from whole brain regions since it can assign high attention weights for important regions. According to the definition in the field of computer vision (Chen et al., 2020), temporal-wise attention can assign weights to different EEG temporal segments collected in one experiment trail. Channel-wise attention can assign a higher weight to a more important feature map and refine feature maps. Spatial-wise attention can identify important feature regions in a single feature map. For example, Woo et al., 2018 propose convolutional block attention module (CBAM), sequentially infers attention maps using channel-wise attention and spatial-wise attention, then the attention maps are multiplied to the input feature map for adaptive feature refinement. To differentiate the three attention methods mentioned above, we use the terminology of EEG channel-wise to describe the attention operation for identifying important EEG channels (i.e., essential nodes) from a single feature map. The weight vector learned by the EEG channel-wise attention helps us to identify the EEG channels which are not important for the specialized network and emphasize the EEG channels which are essential to the specialized network. In addition, we re-term channel-wise attention as specialized network-wise attention to make our study easier to comprehend.

This study addresses the SSVEPs-based frequency recognition task as a multi-category classification problem. It proposes a novel CNN model named group depth-wise convolutional neural network (GDNet-EEG) to execute the task. To overcome the problem of the frequency-specific spatial characteristics might be insufficient to reflect the diversity of brain signals in different brain regions, we construct group depth-wise convolutional filter, which comprises *C* 1D depth-wise convolutional filter, to extract as diverse regional characteristics as possible from raw EEG data. Furthermore, to automatically learn the local connectivity features of specialized networks and the relations between different specialized networks, we propose EEG attention to sequentially infer attention maps along two dimensions (EEG channel and feature map): the former identifies essential brain regions to form a specialized network in a single feature map, and the latter infers important specialized networks across multiple feature maps. More specifically, the GDNet-EEG model is comprised of several group depth-wise convolutional layer. Each layer consists of multiple group depth-wise convolutional filter that employs *C* different 1D depth-wise convolutional filters to process the data outputted by the previous layer. Each depth-wise convolutional filter is separately utilized to process the signal of a single EEG channel and learn regional characteristics originating from different brain regions. *C* denotes the number of EEG channels, i.e., the row number of the feature map in every convolution layer is the same as the EEG channel number. We set *K* group depth-wise convolutional filters to generate *K* feature maps and adopt the same operation in the following convolution layers. Further, the EEG attention is embedded into the GDNet-EEG for learning essential nodes (i.e., significant EEG channel) and meaningful specialized networks (i.e., important feature map). For a feature map generated by a group depth-wise convolution layer, EEG attention first infers attention maps along the EEG channel dimension. Then the attention maps are multiplied by the feature maps for adaptive feature refinement. The refined feature map concerns important brain regions essential to a specialized network. After that, specialized network-wise attention is utilized to give further feature refinement to the different feature maps, highlighting the significance of different specialized networks. The main contributions of this study are depicted as follows:

(1) Unlike the previous studies adopted 1D temporal convolution followed by depth-wise spatial convolutions to extract frequency-specific spatial characteristics, we propose a deep neural network named GDNet-EEG, utilizing group depth-wise convolutional filter to extract regional characteristics from raw EEG data, for SSVEP stimulation frequency recognition. The advantage of using group depth-wise convolutional filter is that it can learn the regional characteristics of the EEG signal and reflect the diversity. The diverse frequency combinations across different EEG channels may be beneficial for EEG-based brain activity classification.
(2) Instead of using DL models to automatically learning the global connectivity feature of all brain regions from BFC matrix, we introduce attention mechanism to identify essential nodes and form specialized connectivity feature of the nodes to improve the performance of SSVEP stimulation frequency recognition. The EEG attention, containing EEG channel-wise attention and specialized network-wise attention, is proposed to identify important EEG channels from a single feature map and recognize important feature map as meaningful specialized networks.
(3) We have used two publicly available SSVEP datasets and their combination dataset consisting of the EEG data of 105 subjects with 40 target characters to validate the model performance of the GDNet-EEG. The related results have been presented to support the correctness of our study.

## 2. Materials and methods

### 2.1. Data description

Two SSVEP datasets (a benchmark dataset for SSVEPs-based BCI (Wang et al., 2016) (benchmark for short) and a large-scale benchmark database toward SSVEP-BCI application (BETA for short) (Liu B. et al., 2020)) and their combination dataset are used to validate the classification performance of the GDNet-EEG model. Each experiment of the benchmark dataset contains six sessions, and each session is comprised of 40 trials. The time length of each trial is 6 s which consists of three parts: gaze shifting of 0.5 s guided by a visual cue, visual stimulation of 5 s, and an offset of 0.5 s followed by the visual stimulation. A target character flickers at a specific frequency on screen in each trial, and the subject is asked to gaze at the flickering character for visual stimulation. The 40 stimulation frequencies are 8–15 Hz with 0.2 Hz strides, and there is a 0.5πphase difference between adjacent frequencies. The EEG data collected in each trial is down-sampled to 250 Hz.

The BETA dataset is similar to the benchmark dataset, and the main difference between them is illustrated as follows. The character matrix layout resembling the traditional QWERTY keyboard is used for the stimulus presentation in the experiment of BETA collection. In contrast, the corresponding layout in the experiment of the benchmark dataset is arranged in a square. The BETA dataset is collected from 70 healthy subjects. Each subject is asked to participate in 4 sessions of the experiment, and each session also consists of 40 trials. The time length of each trial is also comprised of three parts: gaze shifting of 0.5 s guided by a visual cue, visual stimulation of 2 or 3 s, and a rest time of 0.5 s followed by the visual stimulation. Visual stimulation of 2 s and 3 s are given to the first 15 subjects and the remaining 55 subjects, respectively. The EEG data collected in each trial is also down-sampled to 250 Hz.

### 2.2. Data preprocessing

A Chebyshev TypeIfilter filters the EEG signal collected in each trial with cutoff frequencies from 6 to 90 Hz and stopband corner frequencies from 4 to 100 Hz. The multi-channel EEG data collected in one trial is a 2D time series which can be represented by a data matrix *X* of size C*Len, where *C* denotes the number of EEG channels, and *Len* means the signal length of visual stimulation in one-trial EEG record. The record is split into *t* segments {*X_1_, X_2_,…, X_*t*_*}. The size of each segment *X*_*i*_ is C*l, where *l* is the ratio of *Len* and *t*. Each segment *X*_*t*_ has a corresponding classification label *L*_*t*_, and segments collected from the same trial have the same label. The *L*_*t*_ means the target frequency of the visual stimulus given to the subject in the corresponding trial.

### 2.3. GDNet-EEG construction

**FIGURE 1:**
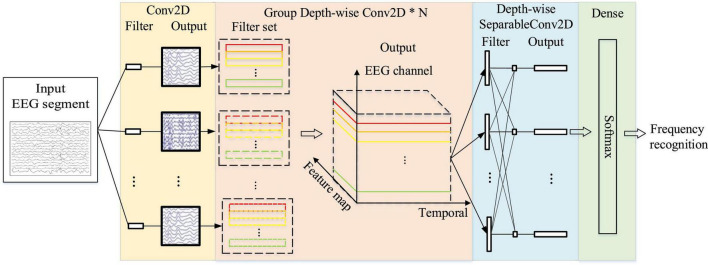
The architecture of the GDNet-EEG model for SSVEP stimulation frequency recognition.

Figure 1 shows the architecture of the GDNet-EEG model, which contains a regular convolution layer, four group depth-wise convolution layers, a depth-wise separable convolution layer, and a dense layer. Note that the regular convolution layer and the depth-wise separable convolution layer are inherited from the EEGNet model to support the feature learning. Considering the pooling operation in the convolution results may cause the loss of meaningful features, we did not add a pooling layer to the GDNet-EEG model. Table 1 shows the specific parameters setting of the GDNet-EEG model. The specific operations of the GDNet-EEG are illustrated as follows:

**TABLE 1:** Specific parameters setting in the GDNet-EEG model, where *C* means the number of EEG channels, *T* denotes the number of time points, and *N* indicates the number of SSVEP stimulation frequencies.

Layer	Layer type	Output size	Hyperparameters
1	Input	(*C*, *Ns*)	
2	Conv2D	(*C*, *Ns*, 64)	$\left\{\begin{array}{c} 1\;\;\;\times \;\;\;17,\;\;64,stride\;1\\ BatchNorm\\ Linear\;Activation \end{array}\right\}\;\;\times \;\;1,\text{ mode}\;=\;\text{same}$
3	Group depth-wise Conv2D	(*C*, *Ns* /16, 64)	$\left\{\begin{array}{c} 1\;\;\;\times \;\;\;17,\;\;64,stride\;2\\ BatchNorm\\ Linear\;Activation \end{array}\right\}\;\;\times \;\;4,\text{ mode}\;=\;\text{same}$
4	Dropout	(*C*, *Ns* /16, 64)	rate = 0.5
5	Depth-wise Conv2D	(1, *Ns* /16, 64)	$\left\{\begin{array}{c} C\;\;\times \;1,\;\;64,stride\;1\\ ELU\;Activation \end{array}\right\}\;\;\times \;\;1,\text{ mode}\;=\;\;\text{valid}$
6	Point-wise Conv2D	(1, *Ns* /16, 16)	$\left\{\begin{array}{c} 1\;\;\;\times \;\;\;1,\;\;16,stride\;1\\ BatchNorm\\ ELU\;Activation \end{array}\right\}\;\;\times \;\;1,\text{ mode}\;=\;\text{same}$
7	Dropout	(1, *Ns* /16, 16)	rate = 0.5
8	Dense	*Nclass*	

#### 2.3.1. Regular convolution layer

This layer aims at generating multiple frequency-specific feature maps which will be fed into the group depth-wise convolution layer for further feature learning. The input of the regular convolution layer is represented by *X*_*i*_∈R^C^*^Ns^ (i.e., a volume of 64 × 50 in the case of *C* = 64, *Ns* = 50 = *T* × = *f*_*s*_ with *T* = 0.2 s and *f*_*s*_ = 250 Hz). As shown in Table 1, 64 convolutional filters are utilized to process the input data, and the size of each filter is set to 1 × 17. Every filter sweeps the temporal and EEG channel dimensions in one stride. This layer is followed by batch normalization and linear activation layer. It utilizes the “SAME” padding mode to pad the input of the convolutional layer if the filter does not fit the input. The output of the layer is represented by *z*_1_∈R^C^*^Ns^^*64^.

#### 2.3.2. Group depth-wise convolution layer

The motivation for using this layer is to learn diverse regional EEG characteristics and deepen the neural network for achieving more abstract EEG features. This layer contains three subparts: group depth-wise convolutional layer, a batch normalization layer, and a linear activation layer. Unlike the traditional depth-wise separable convolutional operation, which utilizes a single depth-wise convolution to convolve the data of each feature map, the group depth-wise convolution employs *C* 1D depth-wise convolutional filters to convolve the EEG data of *C* channels simultaneously. More specifically, we can consider the *C* 1D depth-wise convolutional filters as a filter set that can produce a 2D feature map, and *K* (i.e., *K* = 64) filter sets produce *K* 2D feature maps. The Figure 1 has *K* dashed line frames in black, and each contains a filter set. The long frames with different colors (e.g., red, yellow, blue, or green) represent different depth-wise convolutional filters. The output of the group depth-wise convolution layer is represented by a three-dimensional (3D) feature cube comprised of a feature map, temporal, and EEG channel dimensions. If *l* = 0, layer *l* is the input layer, with the input being EEG fragment *X*_*m*_∈R^C^*^Ns^^*64^. Let *l* (1 ≤ *l* ≤ *N*) be a group depth-wise convolution block. Then, the input of block *l* comprises *m^l–1^* feature maps from the previous block. The output of block *l* consists of *m^l^* feature maps. $Y_{i}^{c,l}$ denotes the row of the *i^th^* feature map in block *l* where *c*∈[1, *C*]. The $Y_{I}^{c,l}$ is computed as follows:


(1) $$Y_{I}^{c,l}=f\left(B_{i}^{c,l}+\sum_{j=1}^{m^{l-1}}K_{i,j}^{c,l}*Y_{j}^{c,l-1}\right)\left(l>=1\right),$$

where $B_{i}^{c,l}$ is bias matric, and $K_{i,j}^{c,l}$ is the convolution filter connecting the *j^th^* feature map in block *l-1* with the *i^th^* feature map in block *l*. After the convolution operation, the leaky rectified linear unit (LeakyReLU) is used as the activation function f(⋅). The *i^th^* feature map is obtained by stacking $Y_{i}^{c,l}$*s* together. Every convolution filter shifts along the temporal dimension by stride *s*1 (i.e., *s*1 = 2). The block *l* is followed by the dropout layer with a dropout rate of 0.5 and adopts the “SAME” padding mode considering the original elements in the layer input. From Table 1, we can see that the filter size (i.e., 1 × 17) equals the size used in the 2D convolutional filter. There are 4 group depth-wise convolution block in the layer, and the final output of the layer is represented by *z*_2_∈R^C^^*(^^Ns/16)^^*64^. Compared with the depth-wise convolution layer in the Compact-CNN to classify 12 categories of SSVEP stimulus frequency, the group depth-wise convolution layer in our model covers the receptive field of the same size. It has a deeper model architecture with fewer parameters which is beneficial for avoiding over-fitting.

#### 2.3.3. Depth-wise separable convolution layer

The motivation for using this layer is to (1) reduce the number of parameters to fit and (2) explicitly decouple the relationship within and across feature maps by first learning a kernel summarizing each feature map individually, then optimally merging the outputs afterward. More specifically, it firstly uses depth-wise spatial convolution in which the kernel shape is C*1 to convolve each 2D feature map into a 1D vector along the temporal dimension of each feature map. Then it utilizes point-wise convolution to combine information across feature map dimensions. The depth-wise spatial convolution layer employs exponential linear unit (ELU)’s nonlinearity and “VALID” padding mode. The filter number of the depth-wise spatial convolution layer is set to 64, and the output of the layer is represented by *z*_3_∈R^(^^Ns/16)^^*64^. It is noteworthy that the depth-wise spatial convolution filter sweeps the data along temporal and EEG channel dimension in one stride and *C* stride, respectively. The point-wise layer is followed by batch normalization and dropout layer. The ELU activation and “SAME” padding mode are adopted in the point-wise convolutional layer. The point-wise convolutional layer employs the convolution filter with size of 1 × 1 to process the data, and the filter number of the point-wise convolution is set to 16 to reduce the number of parameters to fit. The output of the point-wise convolutional layer is denoted by *z*_4_∈R^(^^Ns/16)^^*16^.

#### 2.3.4. Dense layer and the corresponding loss function

The feature maps outputted by the depth-wise separable convolution layer are flattened and concatenated into one vector, fed into the dense layer. It is noteworthy that the GDNet-EEG model only contains one dense layer for avoiding high computation complexity. Let *l* be a dense layer, the identity activation function is utilized as activation function g(⋅), and the output of the *i^th^* unit in layer *l* is computed as follows:


(2) $$Z_{i}^{l}=g\,\left(\sum_{j=1}^{N\,s}w_{i,j}^{l}\,Z_{j}^{l-1}\right),$$

where $w_{i,j}^{l}$, and $Z_{j}^{l-1}$ denote the weights of the *i^th^* unit in layer *l* and the outputs of layer (*l*-1), respectively. The outputs of the dense layer are passed into a softmax function for yielding stimulation frequency recognition results. Thus, the very first input *X*_*i*_ is predicted as $\hat{y}\,\mathrm{argmax}\,s\,\left(Z_{i}^{l}\right)$, where s∈[0,1]*^Nclass^* (i.e., *Nclass* = 40) is the softmax output of the dense layer.

### 2.4. EEG attention module

**FIGURE 2:**
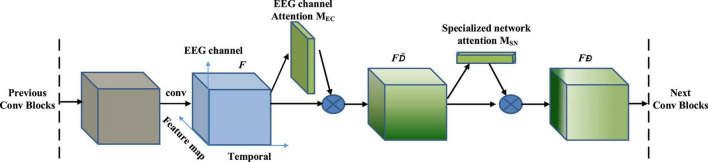
EEG attention integrated with a convolution block in GDNet-EEG.

Figure 2 shows the overall process of the EEG attention module. In the GDNet-EEG, the group depth-wise convolution block output is defined as feature map *F* ∈ R^*C* × *M* × *Len*^, in which *C* represents the number of EEG channels, *M* means the number of feature maps, and Len indicates the length of convolution feature. *F* is fed into the EEG attention module as input. The EEG attention module sequentially infers a 2D EEG channel-wise attention map M_*EC*_ ∈ R^*C* × *M* × 1^ and a 1D specialized network-wise attention vector M_*SN*_ ∈ R^*M* × 1 × 1^. The process of the EEG attention module could be illustrated as:


(3) $${\text{F}}^{{}^{\prime }}=M_{E\,C}\,\left(F\right)\times \text{F},$$

(4) $${\text{F}}^{{}^{\prime \prime }}=M_{S\,N}\,\left(F^{\prime }\right)\times F^{\prime },$$

where F’ is the EEG channel-wise refined feature, calculated by multiplying EEG channel-wise attention map M_*EC*_ and the input feature F. The final output F,” the feature for refining the specialized network, is calculated by multiplying specialized network attention M_*SN*_ and the EEG channel refined feature F’. The final output F” is fed into the next group depth-wise convolution block.

**FIGURE 3:**
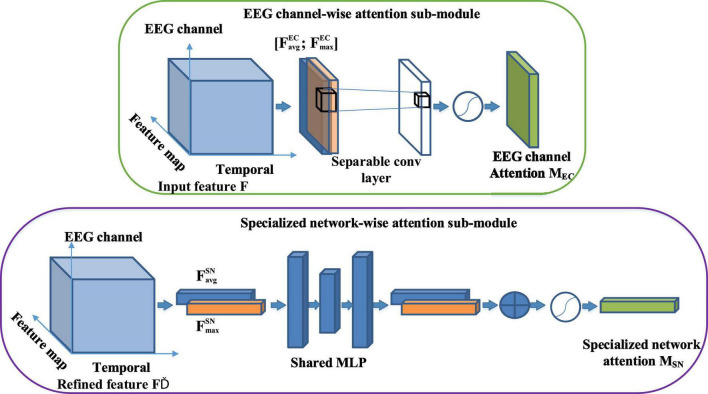
The overall process of the EEG attention module. The module includes two sequential parts: EEG channel-wise attention sub-module and specialized network-wise attention sub-module.

Figure 3 shows the overall process of the EEG attention module. The module includes two sequential parts: EEG channel-wise attention sub-module and specialized network-wise attention sub-module. The EEG channel-wise attention sub-module chooses essential brain regions from each feature map, regarded as a specialized network. The specialized network-wise attention sub-module acts on the feature map refined by the EEG channel-wise attention and generates an attention vector to represent the importance of different specialized networks. As the top part of Figure 3 shows, we have generated the EEG channel-wise attention map along the feature map dimension. Every feature map generated by the previous convolution layer is downsampled along the convolution feature dimension using both average and maximum pooling. Every feature map is down-sampled into a 1D vector whose length is the same as the EEG channel number. The data representation of the average-pooled feature $F_{a\,v\,g}^{\text{EC}}$∈ R^*C* × *M* × 1^ and max-pooled feature $F_{\text{max}}^{\text{EC}}$∈ R^*C* × *M* × 1^ are 2D matrix, in which the row represents the EEG channel, and the column means feature map. We stack the $F_{\text{avg}}^{\text{EC}}$ and $F_{\text{max}}^{\text{EC}}$ together as the input of a separable convolution layer, which uses M 1*1 convolution filters to separately convolve the pooled feature stack along the EEG channel axis and generate M vectors. Every vector is passed into a sigmoid function to assign attention weight for EEG channels in every feature map. M attention weight vectors constitute the 2D EEG channel-wise attention map M_*EC*_. The EEG channel-wise attention map is computed as follows:


$$M_{E\,C}\,\left(F\right)=\sigma \,\left(f^{M;1*1}\,\left(\left[A\,v\,g\,P\,o\,o\,l\,\left(F\right);M\,a\,x\,P\,o\,o\,l\,\left(F\right)\right]\right)\right)=$$

(5) $$\,\sigma \,\left(f^{M;1*1}\,\left(\left[F_{a\,v\,g}^{E\,C};F_{m\,a\,x}^{E\,C}\right]\right)\right),$$

where σ means the sigmoid function and*f*^*M*;1*1^ denotes a separable convolution network.

As the bottom part of Figure 2 illustrates, the input of the specialized network-wise attention is the feature maps refined by the EEG channel-wise attention sub-module. These are the dot multiplication results of the 2D EEG channel-wise attention map M_*EC*_ and the original feature map F. The feature maps refined by the EEG channel-wise attention sub-module are pooled by using two pooling operations: average-pooled feature $F_{a\,v\,g}^{\text{SN}}$∈ R^*M* × 1 × 1^ and max-pooled feature $F_{\text{max}}^{\text{SN}}$∈ R^*M* × 1 × 1^. The two vectors are forwarded separately to a shared network composed of a multi-layer perceptron (MLP) with one hidden layer to produce two refined pooled vectors. After the shared network is applied to each descriptor, we merge the output feature vectors using element-wise summation. The specialized network-wise attention is computed as follows:


$$M_{S\,N}\,\left(F\right)=\sigma \,\left(\text{MLP}\,\left(\text{AvgPool}\,\left(F^{{}^{\prime \prime }}\right)\right)+M\,L\,P\,\left(M\,a\,x\,P\,o\,o\,l\,\left(F^{{}^{\prime \prime }}\right)\right)\right)$$

(6) $$=\sigma \,\left(W_{1}\,\left(W_{0}\,\left(F_{a\,v\,g}^{S\,N}\right)\right)+W_{1}\,\left(W_{0}\,\left(F_{m\,a\,x}^{S\,N}\right)\right)\right),$$

where σ denotes the sigmoid function, *W*_0_ and *W*_1_ are the MLP weights shared for average-pooled vector $F_{a\,v\,g}^{S\,N}$ and max-pooled vector $F_{m\,a\,x}^{S\,N}$.

## 3. Results

### 3.1. Experimental setup

The EEG data collected during the visual stimulation period is kept. To split the raw EEG data collected in each session into EEG segments, we remove the EEG data collected during the gaze shifting of 0.5 s guided by a visual cue and an offset of 0.5 s followed by the visual stimulation. The benchmark dataset contains 8,400 trials and 40 categories, and the time length of the flickering visual stimulation in each trial is 5 s. The BETA dataset consists of 11,200 trials and 40 categories. For the first 15 participants and the remaining 55 participants in the BETA dataset, the time length of the flickering visual stimulation in each trial is 2 and 3 s, respectively. For generating the input of the GDNet-EEG and other comparison models, we first extract the raw EEG data of each trial of the two datasets to form data samples and assign the corresponding flickering character as the label to each data sample. Further, we apply a sliding window with the step of *ratio* × 250 on each data sample and generate the final input samples in a non-overlapping manner. For example, assuming the *ratio* equals 0.4, the data shape of each input sample is 100u*N*_*c*_, and the *N*_*c*_ denotes the number of EEG channels (i.e., 64).

Because longer EEG segments contain more information about brain activity, the model performance for target frequency identification can be improved by increasing the segment length *T*. Considering this fact, we investigated the impact of segment length *T* ranges [0.2, 0.4, 0.6, 0.8, and 1.0] on model performance. More specifically, when the number of data points of each input sample is 50, meaning the *ratio* is set to 0.2, and segment length *T* representing the time length of each input sample is 0.2 s, the total number of input samples of the combination dataset for training and testing models is 366,000. The models are trained with a batch size of 64, and mini-batch gradient descent and Adam optimizer with a learning rate of 0.001 are used to optimize the model parameters. An early-stop training strategy is adopted to train the models. Ten-fold cross-validation is applied to divide the dataset into training data and testing data, and the average classification accuracy (ACC) rate, sensitivity (SEN), and specificity (SPE) and the corresponding standard deviation (STD) of them are employed as model performance metrics. The above metrics are calculated using the following formulas:


(7) ACC=(TP+TN)/(TP+FP+FN+TN),

(8) SEN=TP/(TP+FN),

(9) SPE=TN/(TN+FP),

where TP denotes true positives, TN denotes true negatives, FP denotes false positives, and FN denotes false negatives.

### 3.2. Model training and further details

The GDNet-EEG and other comparable models are implemented by Pytorch and trained with a Tesla A100 GPU. The GDNet-EEG model is initialized by sampling the network weights from Gaussian distribution with 0 mean and 0.01 variance. Categorical cross-entropy is used as the loss function to train the model by comparing the probability distribution with true distribution. More specifically, the EEG data collected in one trial is represented by (*X*, *Y*), where *X*∈R^C^*^Len^ and *Y*∈R*^Nclass^*. As mentioned above, *X* is split into *t* segments {*X_1_, X_2_,…, X_*t*_*} and segments collected from the same trial have the same label *Y*. To train the GDNet-EEG, we select the EEG signal of *D*_*b*_ trials as a batch of data to train the model in each iteration. The loss function of the categorical cross-entropy is computed as follows:


(10) $$\frac{-1}{t*D_{b}}\,\sum_{i=1}^{t*D_{b}}\sum_{j=1}^{N\,c\,l\,a\,s\,s}y_{i\,j}\,l\,o\,g\,\left(s_{i\,j}\right)+\lambda \,{\left|w\right|}^{2},$$

where λ (i.e., λ = 0.001) denotes the constant of the L2 regularization. *s_*ij*_*∈[0,1]*^Nclass^* and *y_i_* represent softmax output for the input segment *X*_*i*_ and the corresponding frequency label of the input segment *X*_*i*_, respectively. *w* means the weights of the GDNet-EEG model. The GDNet-EEG model is trained by two stages: the first stage is trained by the benchmark dataset and the second stage is trained by the BETA dataset. Note that the second stage re-initializes the network with the weights trained by the first stage and fine-tunes the weights to fit the data distribution of the BETA dataset. The model training strategy originates from the consideration of inter-dataset statistical variations.

### 3.3. Comparison baselines

Five kinds of CNN models are reproduced as baseline approaches for result comparison. To perform the SSVEPs-based stimulation frequency recognition task, we reconstruct the output layer of these models to distinguish 40 target stimulation frequencies. The simplified description of the baseline approaches is depicted as follows:

EEGNet (Lawhern et al., 2018): The network starts with a temporal convolution to learn frequency filters and then uses depth-wise convolution to learn frequency-specific spatial filters. The depth-wise convolution combines all EEG channels to obtain a better frequency pattern.

Compact-CNN (Waytowich et al., 2018): The network is a variant of the EEGNet for classifying the SSVEP signals. Unlike the EEGNet, the dense layer of the Compact-CNN does not adopt the max-norm constraint function to the kernel weights matrix.

DeepConvNet (Schirrmeister et al., 2017): The model is a deep convolution network for end-to-end EEG analysis. It is comprised of four convolution-max-pooling blocks and a dense softmax classification layer. The first convolutional block is split into a first convolution across time and a second convolution across space (electrodes). The following blocks utilize standard convolution operation with a large filter whose width is equivalent to the number of feature maps.

Shallow ConvNet (Schirrmeister et al., 2017): The network is a shallow version of the DeepConvNet and contains one convolution-max-pooling block and a dense softmax classification layer. Compared with the deep ConvNet, the temporal convolution of the shallow ConvNet adopts a larger kernel size. After the two convolutions of the shallow ConvNet, a squaring nonlinearity, a mean pooling layer, and a logarithmic activation function followed.

Convolutional correlation analysis (Li et al., 2020): The network consists of a signal-CNN branch and a reference-CNN branch. The former is comprised of three convolutional layers, and the latter contains two convolutional layers. The output of the two branches is fed into the dropout layer for regularization. A correlation layer is followed by the dropout layer for calculating the correlation coefficients of the output of the two branches. A dense layer and softmax activation function is applied as the final classification layer.

FB-SSVEPformer (Chen et al., 2022c). This is the first Transformer-based deep learning model for SSVEP classification. The frequency spectrum of the SSVEP signals is extracted by filter bank technology and fed into SSVEPformer, which further learns spectral and spatial characteristics by self-attention mechanism for final frequency classification.

Filter bank CCA (Chen et al., 2015). This method tries to make use of harmonic SSVEP components to enhance the CCA-based frequency detection. By incorporating the fundamental and harmonic SSVEP components in target identification, the method significantly improves the performance of the SSVEP-based BCI.

### 3.4. Ablation studies

On the one hand, we design a comparison experiment to compare the classification performance of the GDNet-EEG model and its variations. The motivation of designing this comparison experiment is to validate the main innovations of our model, such as group depth-wise convolution and EEG attention module. On the other hand, the effect of EEG channel number on the model performance is also validated for demonstrating whether our model can recognize more informative SSVEP features from the signal of multiple EEG channels or not.

#### 3.4.1. Comparison results between the GDNet-EEG model and its variations

The main innovation of our model mainly includes two aspects: (1) GDNet-EEG is a deep convolution architecture using a group depth-wise convolutional filter to extract as diverse regional characteristics as possible from raw EEG data. (2) EEG attention consisting of EEG channel and specialized network-wise attention is proposed to refine EEG feature of single EEG channel and recognize specialized networks to improve the model performance of SSVEPs-based target stimulation frequency recognition. To validate the model performance of the GDNet-EEG affected by the above two aspects, we design the following models: (1) we adopt a regular convolutional filter to substitute the group depth-wise convolutional filter in the GDNet-EEG; (2) we implement a shallow version of the GDNet-EEG, comprised of two group depth-wise convolutional layers; (3) we remove the EEG attention module of the GDNet-EEG; (4) the EEG channel-wise attention is removed from the GDNet-EEG; (5) the specialized network-wise attention is removed from the GDNet-EEG; (6) Instead of using EEG attention module, we embedded CBAM block into the GDNet-EEG model for refining the feature maps learned by the group depth-wise convolution layer We use model 1 model 6 to denote the five models for simplification.

**FIGURE 4:**
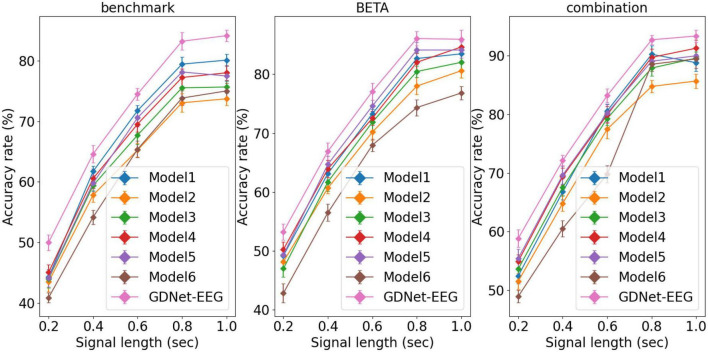
Average classification accuracies obtained by GDNet-EEG and model 1∼model 6 over 10-fold cross-validation. Error bars indicate standard errors.

The model performance affected by the signal length of the input sample is investigated. Figure 4 gives average classification accuracies obtained by the GDNet-EEG and model 1 model 6 over 10-fold cross-validation, and error bars indicate standard errors. The figure shows that the GDNet-EEG outperforms other models in classification accuracy across the three datasets in various signal lengths. As the signal length increases, the classification accuracy of different models shows an upward trend. This result shows that the EEG signal with a longer time length contains a more apparent characteristic pattern, which facilitates the deep learning models to generate more accurate decisions. Especially in the signal length of 1 s, the GDNet-EEG model achieves the highest classification accuracy of 84.11, 85.93, and 93.35% on the benchmark, BETA, and combination datasets, respectively. The models trained on the combination dataset obtained better model performance than the models trained on the benchmark dataset and BETA dataset, which may be attributed to the impact of dataset size on the deep learning model. Compared with the model 1 which is implemented by a regular convolutional filter, the GDNet-EEG obtains better classification accuracy, indicating the superiority and rationality of the group depth-wise convolution layer. The shallow GDNet-EEG (model 2) achieves the lowest accuracy, indicating the deep layer structure might provide an accuracy increment for the GDNet-EEG. The superiority of the EEG attention is also validated by comparing model 3 model 5 with the classification accuracy of the GDNet-EEG. More specifically, the classification rate of the model 3 is lower than the classification rate of our model, as well as the classification performance of model 4 or model 5 is also worse than the classification performance of the GDNet-EEG, demonstrating the EEG attention module can improve the classification performance of the GDNet-EEG. The comparison results between classification rate of model 4 and model 5 indicate the specialized network-wise attention seems to be capable of better boosting the classification performance of our model. By comparing the classification performance of model 6 with the classification performance of the GDNet-EEG, we can know the EEG attention module might be more suitable for refining representational EEG feature and improve the model performance for target frequency identification.

#### 3.4.2. Effect of EEG channel number on the model performance

Note that the EEG channel location is arranged by international 10-10 EEG system. Although previous studies demonstrated the EEG channels that are placed over the occipital and parietal regions provide perhaps the most informative SSVEP signals, we want to validate the effectiveness of our approach on using the data of varying number of EEG channel. Table 2 gives the classification results (ACC, SPE, SEN, and their corresponding STDs) of our model is reported versus varying number of channels and 1.0 s of stimulation. We conducted five experiments to validate the effect of varying number of EEG channel on the model performance, the channel number and the corresponding channel name are given as follows:

• three EEG channels (labeled by O1, Oz, and O2) that are placed over the occipital (O) regions;
• six EEG channels (labeled by O1, Oz, O2, POz, PO3, and PO4) that are placed over the occipital and parietal- occipital (PO) regions, it is noteworthy that PO denotes the EEG channel placed between occipital and parietal regions;
• on the basis of the six EEG channels, we add another three EEG channels that are placed over PO regions, the nine EEG channels are labeled by O1, Oz, O2, Pz, PO3, PO5, PO4, PO6, and POz;
• thirty-two EEG channels that are placed over occipital, parietal, central, and central-parietal regions.
• Sixty-four EEG channels are placed over all brain regions.

**TABLE 2:** Classification results (ACC, SPE, SEN, and their corresponding STDs) of our model is reported versus varying number of channels and 1.0 s of stimulation.

Channel number	Benchmark			BETA			Combination		
	ACC (%)	SPE (%)	SEN (%)	ACC (%)	SPE (%)	SEN (%)	ACC (%)	SPE (%)	SEN (%)
3	65.32 ± 1.96	68.95 ± 2.12	63.58 ±	70.52 ± 1.74	69.72 ± 3.26	72.56 ± 2.51	86.16 ± 2.07	83.47 ± 1.85	88.73 ± 2.36
6	68.89 ± 2.52	70.32 ± 1.73	65.49 ±	72.46 ± 1.38	69.89 ± 2.79	73.85 ± 1.86	86.73 ± 1.96	84.59 ± 2.20	89.39 ± 1.82
9	75.28 ± 1.15	78.64 ± 1.58	73.24 ±	76.57 ± 2.21	74.87 ± 2.58	77.31 ± 2.70	91.27 ± 1.47	89.76 ± 1.63	92.26 ± 2.18
32	80.19 ± 1.09	81.79 ± 1.17	79.37 ±	82.91 ± 1.93	79.41 ± 2.90	83.46 ± 1.93	91.52 ± 2.15	89.50 ± 2.37	91.87 ± 2.60
64	84.11 ± 1.28	85.27 ± 0.93	83.81 ± 1.70	85.93 ± 1.36	83.26 ± 2.14	86.97 ± 2.36	93.35 ± 1.59	91.24 ± 1.54	94.12 ± 1.67

The results demonstrate that there is an increasing tendency of the classification metrics of our approach as the EEG channel number increases, indicating the data collected from all EEG channels can help to improve the model performance. In addition, it is noteworthy that based on the combination dataset, the classification metrics of 9 EEG channels are close to the classification metrics of 32 EEG channels while lower than the classification metrics of 64 EEG channels. This result indicates the EEG channels that are placed over the occipital and parietal regions might provide the most informative SSVEP signals while other channels might be informative as well.

### 3.5. Comparison studies

**FIGURE 5:**
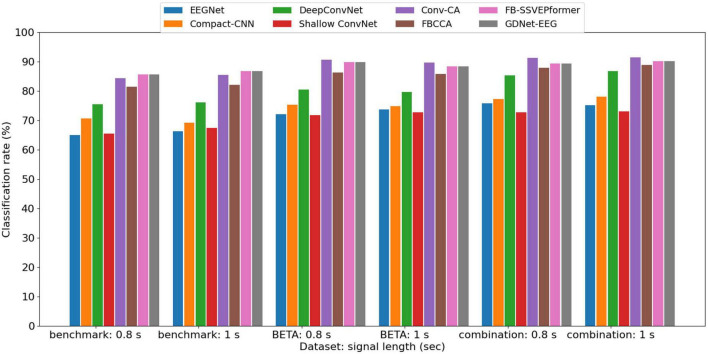
The average classification accuracies obtained by GDNet-EEG and five other models over 10-fold cross-validation using a signal length of 0.8 and 1 s.

The ablation study shows that the GDNet-EEG model achieves the best classification accuracies based on the three datasets with the input sample length of 0.8 and 1 s. To further validate the model performance of the GDNet-EEG, we present average classification accuracies obtained by GDNet-EEG and five other models over 10-fold cross-validation using the signal length of 0.8 and 1 s. Figure 5 shows that the average classification accuracies of the other five model baselines trained on a combination dataset decreased from 1.96 to 18.2% compared to the GDNet-EEG. It indicates that the GDNet-EEG can produce more robust features than existing EEG-oriented deep learning methods and improve the discriminability between different stimulation frequencies. Compare with FB-SSVEPformer, our model achieves better classification rate based on the combination dataset, indicating the superiority of the GDNet-EEG based on the dataset with larger scale. In addition, the average classification accuracies of the FBCCA are lower than the classification accuracies of the GDNet-EEG model across the three EEG datasets, while the Conv-CA trained on the benchmark and BETA datasets outperformed the GDNet-EEG in average classification accuracies. Since the technical route of the Conv-CA and the GDNet-EEG is different, it gives us a cue for adapting the model architecture of the GDNet-EEG by integrating the CCA method to discriminate stimulation frequencies.

## 4. Discussion

Glaucoma is a common eye condition caused by a damaged optic nerve and can lead to vision loss if not diagnosed and treated early. The SSVEPs-based BCI application can generate brain signals when human looks at something flickering. If a patient has a blind area in a region, the signals extracted from these stimuli are weak, and it is reflected on the visual response map. That is, the patient cannot accept the stimulation from the flickering object at the field of vision loss occurred. Thus, the SSVEPs-based BCI application, e.g., visual speller, can diagnose glaucoma (Lin et al., 2015; Nakanishi et al., 2017; Khok et al., 2020). Based on SSVEPs-based BCI application, accurate glaucoma diagnosing requires effective EEG analysis methods to discriminate stimulation frequencies. Machine learning methods, especially deep learning, can achieve high accuracy in EEG-based classification tasks. However, most EEG-oriented deep learning methods focused on applying existing techniques to the EEG-based brain activity analysis task rather than proposing new ones specifically suited to the domain (Rasheed and Extraction, 2021). The standard well-known network architectures were designed for the data collected in natural scenes (e.g., natural images) and did not consider the EEG-based brain activity’s peculiarities. Therefore, research must understand how these architectures can be optimized for SSVEPs-based classification tasks.

The peculiarities of EEG-based brain activity at least include the following two aspects: regional characteristics and network characteristics. The former can be represented by the temporal and spectral features of the signal generated from a single brain region. The BFC can represent the latter *via* learning all brain regions’ global and local connectivity features. Although many existing studies extract temporal, spectral, and spatial features to represent the regional and network characteristics and feed them into deep learning models for generating decision results (Rocca et al., 2014; Amin et al., 2019; Su et al., 2020), they are not end-to-end deep learning frameworks. Convolution operation using the 1D convolutional filter is the priority choice for building the end-to-end deep learning framework for SSVEPs-based BCI applications (Waytowich et al., 2018). Unlike the previous studies using the regular 1D convolutional filter to learn EEG features, we utilize group depth-wise convolution operations containing a set of 1D convolutional filters and use each filter to convolve the data of the corresponding single brain region. An attention mechanism is adopted to identify important EEG channels from a single feature map and recognize significant feature maps as specialized brain networks.

An ablation study and comparison study are implemented to validate the performance of our proposed method in discriminating stimulation frequencies. From the experiment results described in Figures 4, 5 we can conclude that the average classification accuracies achieved by the models trained on the combination dataset are better than the average classification accuracies of the models trained on the benchmark and BETA datasets. The average classification accuracies obtained *via* the models trained on the BETA dataset are better than the models trained on the benchmark dataset. The reason can be explained from the aspect of deep learning model performance affected by the dataset size. As we know, insufficient training data can lead to poor performance of deep learning models. Small training and testing datasets will result in underfitting the deep learning model, generating an optimistic and high variance estimation of model performance. By observing the experiment results of the ablation study, we can see an upward trend of average classification accuracies along with the signal length increasing. This result coincides with the experiment result of other studies (Li et al., 2020; Guney et al., 2021), which indicates better classification accuracy can be obtained by lengthening the stimulation duration (i.e., signal length of input sample). In addition, the comparison results between the average classification accuracies obtained by the GDNet-EEG using a regular 1D convolutional filter. Additionally, our method demonstrates the superiority of the group depth-wise convolution operation. Compared with EEGNet and Compact-CNN, our model’s group depth-wise convolution layer covers the receptive field of the same size and has a deeper model architecture with fewer parameters. The higher classification accuracies achieved by our model indicate that the architecture of our model can capture more robust EEG features to discriminate stimulation frequencies. The ablation study also validates that using an attention mechanism can improve the classification accuracies of models in discriminating different stimulation frequencies.

Our proposed GDNet-EEG has three potential improvement directions: (1) This study is a pilot study for glaucoma diagnosing by implementing an effective deep learning method for SSVEPs-based stimulation frequency discrimination. The datasets used in this study are collected from healthy participants. Collecting an SSVEP dataset from glaucoma patients is a feasible route for making our method more available in SSVEPs-based BCI application of early glaucoma diagnosis. (2) Inspired by the method of using CCA to discriminate stimulation frequencies, we plan to use a self-attention mechanism (e.g., Transformer model) (Vaswani et al., 2017) to calculate how similar between stimulation signals and reference signals and utilize the similarity to generate more robust EEG feature for discriminating stimulation frequencies. (3) Although the experimental results have demonstrated that group depth-wise convolution and EEG attention facilitates the GDNet-EEG to achieve promising classification performance in discriminating SSVEPS-based stimulation frequencies, this result may be unable to provide strong support for clinical treatment that is associated with EEG biomarkers. Because DL methods are essentially black boxes, we require novel methods to open the box and visualize the feature learned by the DL model. To this end, an emerging technique known as explainable artificial intelligence (AI) (Gunning et al., 2019) enables the understanding of how DL methods work and what drives their decision-making. We plan to use the explainable AI method to visualize the critical brain regions and significant specialized networks and further validate our method’s performance.

## 5. Conclusion

In this study, we propose a novel deep learning model named the GDNet-EEG, which is tailored to learn regional characteristics and network characteristics of EEG-based brain activity to perform the SSVEPs-based stimulation frequency recognition task. The group depth-wise convolution is proposed to extract temporal and spectral features from the EEG signal of each brain region and represent regional characteristics as diverse as possible. Based on the output of the group depth-wise convolutional layer, EEG attention consisting of EEG channel-wise attention and specialized network-wise attention is designed to identify essential brain regions and form significant feature maps as the specialized brain functional networks. The experiment results demonstrate that our method outperforms the existing deep learning models tailored to process EEG data on two publicly SSVEPs datasets (large-scale benchmark and BETA dataset) and their combined dataset. Our approach could be potentially suitable for providing accurate stimulation frequency discrimination and being used in the early glaucoma diagnosis using SSVEP signals.

## Data availability statement

The original contributions presented in the study are included in this article/supplementary material, further inquiries can be directed to the corresponding authors.

## Author contributions

ZW and WD contributed to the conception and design of the study and drafted the manuscript. WC and ML performed the data analysis. RZ provided technique and writing guidance. All authors contributed to the article and approved the submitted version.
